# Cost trends of potentially inappropriate medications among older adults between 2012 and 2021 in Quebec, Canada: a population-based repeated cross-sectional study

**DOI:** 10.1186/s12877-025-06235-7

**Published:** 2025-08-02

**Authors:** Magalie Gagnon, Jason Robert Guertin, Caroline Sirois, Marc Simard, Benoît Cossette, Marie-Eve Gagnon

**Affiliations:** 1 Faculté de pharmacie, Université Laval, Québec, Québec Canada; 2 Département de médecine sociale et préventive, Université Laval, Québec, Québec Canada; 3 Centre de recherche du CHU de Québec, Université Laval, Québec, Québec Canada; 4 Centre d’excellence sur le vieillissement de Québec, VITAM – Centre de recherche en santé durable, Québec, Québec Canada; 5 Institut national de santé publique du Québec (INSPQ), Québec, Québec Canada; 6 Faculté de médecine et des sciences de la santé, Université de Sherbrooke, Sherbrooke, Québec Canada; 7 Present Address: Département des sciences de la santé, Université du Québec à Rimouski (UQAR), 1595 Bd Alphonse-Desjardins, Lévis, Québec G6V 0A6 Canada

**Keywords:** Potentially inappropriate medication, Costs, Trend, Older adults, Public expenditures

## Abstract

**Background:**

Potentially inappropriate medications (PIMs) are frequent in older adults, contributing to hospitalizations, adverse events, and healthcare burden. We aimed to estimate direct PIM cost trends from 2012 to 2021 among older women and men in Quebec, Canada.

**Methods:**

Using medico-administrative data, we assessed direct costs paid by the public insurer (medication cost and professional fee, excluding out-of-pocket payments by individuals) of PIMs claimed by adults ≥65 years covered by the public drug plan. Costs for 16 PIM classes, identified using 2015 and 2019 Beers criteria, were calculated and stratified by sex and age group (65-74, 75-84, ≥85) for each fiscal year. We assessed the proportion of PIMs among all medication expenditures. We computed average costs/enrollee and usage prevalence for the costliest PIM classes. Trends were estimated using univariate linear regression with 95% confidence intervals.

**Results:**

We found a non-statistically significant decrease in total PIM claim costs, from $206 million in 2012 to $186 million in 2021 (trend: -2.9[-17.4; 11.6]), representing 5.4% of medication expenditures for adults ≥65 in 2021. The reduction in total costs was more accentuated in women, whose annual costs were higher than those of men in all age groups. Average cost/enrollee decreased from $179 to $119 (trend: -7[-19; 5]), with a drop from $216 to $142 for women and $132 to $92 for men. Costs/enrollee were higher in 75-84 and ≥85 age groups. Costliest PIM classes included proton-pump inhibitors, benzodiazepines, antipsychotics, antidepressants, estrogens (women), and hypoglycemic agents (men). Cost trends did not always follow prevalence trends for these PIM classes.

**Conclusion:**

PIM costs among older adults slightly decreased from 2012 to 2021. Appropriate prescribing and deprescribing appear crucial for reducing these costs. Further research should focus on estimating the societal impact and the cost-effectiveness analysis of deprescribing initiatives and other regulatory measures.

**Supplementary Information:**

The online version contains supplementary material available at 10.1186/s12877-025-06235-7.

## Introduction

With the population aging, the prevalence of chronic diseases is rising, leading to an increase in medication use and an important financial burden [1–6]. In 2021–2022, 61% of public drug plan expenses in Quebec, Canada, were allocated to enrollees aged ≥ 65, totaling $2.59 billion, while this age group represents 41% of enrollees [7]. Moreover, a high number of prescribed medications is a known factor contributing to potentially inappropriate medications use (PIMs) [3, 4, 8].

PIMs are defined as medications that pose more risks than benefits when used among older adults [9, 10]. These medications can lead to adverse effects and hospitalizations, while safer or more effective alternatives exist [4, 11]. Many tools are available to identify PIMs, but the American Geriatrics Society Beers criteria is among the most widely used in North America, particularly with medico-administrative data. These guidelines list medications to avoid among adults ≥ 65 years [9, 10]. In Canada, nearly half of the older population is estimated to use at least one PIM, with a higher prevalence among women than men [3, 4].

In 2013, PIM direct costs (medication cost and dispensing fees) in Canada were estimated at $419 million including costs paid by the public drug plan, and out-of-pocket payments by individuals [12]. Over the past decade, various initiatives promoting appropriate prescribing and deprescribing have been implemented [13, 14], potentially influencing societal PIM costs [15]. In fact, inflation-adjusted PIM costs appear to have decreased by 33% from 2013 to 2021 [16]. However, as these results are based on projections, the actual direct costs, which are very important for health authorities, remain unknown, as well as their indirect costs including associated public healthcare services uses. Analyzing actual PIM cost patterns could provide insight into the use of public funds for controversial medications, potentially justifying investment in public awareness and deprescribing initiatives. As a first step toward a broader understanding of the societal impact of PIMs, we aimed to estimate trends in the actual direct costs of PIMs over a 10-year period (2012–2021) among women and men aged ≥ 65 covered by the Quebec’s public drug plan, from the public payor perspective.

## Methods

### Study design and setting

We used a retrospective repeated cross-sectional design involving older adults in the province of Quebec, in Canada. The direct PIM costs were estimated from the public payor’s perspective, the *Ministère de la Santé et des Services sociaux*.

### Data source

Using data from the public drug plan, we included all enrollees ≥ 65 years in Quebec, representing around 90% of older Quebecers [17], excluding the remaining population covered by private drug insurance plans or receiving medication through health and social service institutions (e.g., long-term care facilities). We used cost data from medication claims dispensed in community settings for each fiscal year from April 1 st to March 31 st, covering the 10-year period from 2012 to 2021. Data were extracted by sex and age groups (65–74, 75–84 and ≥ 85) for each year. Data on PIM costs, including medication costs and dispensation fees, were obtained from the *Régie de l’assurance maladie du Québec* (RAMQ) which manages public drug plan data. Medication claims included those on the standard RAMQ medication list and those reimbursed through the exceptional medication measure (reimbursement for certain medications under predefined clinical criteria [18]) and patient exception measure (reimbursement for certain medications for specific patients [19], e.g., Z-drugs). We considered all medication claims for which the public drug plan contributed, whether individuals were partially or fully covered for each fiscal year. Direct costs included medication costs and professional fees paid by the public insurer, excluding the portion that individuals are required to pay out-of-pocket with each dispensation (deductibles and coinsurances) [20]. From this database, the indirect costs of PIMs were not available.

Publicly available RAMQ information was used to obtain the number of individuals covered by the public drug plan for each calendar year (January 1 st to December 31 st). We extracted these official data from the Tables AM.02 on the St@tRAMQ website [21]. Data were extracted by sex and age groups (65–74, 75–84 and ≥ 85). The number of individuals corresponded to full-time equivalent community-dwelling patients.

Costs were converted in 2024 Canadian dollars, using the Canadian Consumer Price Index (all basket components) on April 1 st, 2024 [22].

### Variables

PIMs were identified using Table 2 of the 2015 version [9] of the American Geriatrics Society’s Beers criteria for the years 2012 to 2018, and the 2019 version [10] for the period from 2019 to 2021. We adapted the criteria to the Canadian context with the commercialized medications. Specifically, we retained medication belonging to classes considered potentially inappropriate (e.g. antipsychotics) that were available on the Canadian market, even if they were not commercialized in the United States, and we excluded those listed in the original criteria that were not available in Canada. Table S1 (supplementary files) lists all included medications by common denomination code [23] corresponding to the fifth Anatomical Therapeutic Chemical classification system level [24]. We excluded parenteral forms of antipsychotics, antispasmodics, benzodiazepines, metoclopramide and proton-pump inhibitors (PPIs), as these forms are not typically used in community settings due to their complexity and the heightened level of surveillance they require.

The number of claims and associated costs for each PIM were extracted and aggregated into 16 classes inspired by the 2015 and 2019 Beers criteria. Some medication classes were grouped under broader categories (hypoglycemic agents and other gastrointestinal drugs) to ensure sufficient category sizes. The final categories included: analgesic agents, antidepressants, antiparkinsonian drugs, antipsychotics, antispasmodics, barbiturates, benzodiazepines, cardiovascular drugs, estrogens, first-generation antihistamines, hypoglycemic agents (including sulfonylureas and rapid insulins without concomitant basal or long-acting insulin), muscle relaxants, non-benzodiazepine hypnotics, oral nonsteroidal anti-inflammatory drugs (NSAIDs), other gastrointestinal drugs, and PPIs. Since there were no claims for the other central nervous system drugs and desiccated thyroid drugs throughout the study period, these classes were excluded from the analyses.

### Statistical analysis

We assessed the proportion of PIMs among all medication expenditures in adults ≥ 65 years. Total and average costs per enrollee for all PIM classes were estimated for each fiscal year and stratified by sex and age, where age was defined at the fiscal year level. Average costs per enrollee were calculated by dividing the total costs for each fiscal year by the full-time equivalent population for the corresponding calendar year. Average costs per enrollee and prevalences were computed for the five most expensive classes, further stratified by sex and age from 2012 to 2021. We performed univariate linear regression to estimate PIM cost trends across each fiscal year over the study period (2012–2021), with 95% confidence intervals.

As a sensitivity analysis, we restricted our examination of cost trends to the 2012 to 2018 period, which corresponds to the application of the 2015 Beers criteria version, before the implementation of the 2019 version. This analysis was performed to account for potential differences due to changes in criteria. We did not analyze the subsequent period (2019–2021) due to the limited data. We also calculated costs in nominal values, along with trend slopes and confidence intervals. All analyses were conducted using Microsoft Excel for Microsoft 365 (2407 version).

## Results

Table 1 presents data on full-time enrollees ≥ 65 years in the Quebec public drug plan for each year. The total number of enrollees went from 1,150,383 in 2012 to 1,558,353 in 2021, reflecting a 35.5% relative increase (30.9% for women; 41.3% for men). Throughout the entire timeframe, women and those in the 65–74 age group consistently represented a larger proportion of the population.

**Table 1:** Number of Quebec public drug plan enrollees aged ≥65 by year, sex and age group (2012-2021)

	**Age group years)**	**Number of individuals**^a^									
		**2012**	**2013**	**2014**	**2015**	**2016**	**2017**	**2018**	**2019**	**2020**	**2021**
**Women**	65-74	341,308	356,589	371,060	384,629	398,388	411,318	423,486	435,898	449,178	459,872
	75-84	214,614	216,054	218,967	221,851	226,378	231,942	239,659	249,217	259,859	271,765
	≥ 85	92,313	95,920	99,435	102,424	106,498	109,962	112,006	113,808	115,632	117,164
	Total ≥ 65	648,235	668,564	689,461	708,905	731,265	753,223	775,151	798,922	824,670	848,800
**Men**	65-74	303,588	318,082	331,480	344,667	358,069	371,321	383,830	397,050	410,522	422,166
	75-84	156,784	160,151	164,706	168,759	174,614	181,377	189,896	199,759	210,519	222,332
	≥ 85	41,777	44,563	47,338	49,995	53,258	56,431	58,967	61,157	63,286	65,054
	Total ≥ 65	502,148	522,797	543,524	563,420	585,942	609,128	632,693	657,966	684,327	709,553
**Overall**		1,150,383	1,191,361	1,232,985	1,272,326	1,317,207	1,362,351	1,407,844	1,456,888	1,508,997	1,558,353

Data were extracted from tables AM-02 on the St@tRAMQ website

^a^Full time equivalent population per calendar year from January 1^st^ to December 31^st^

**Fig. 1:**
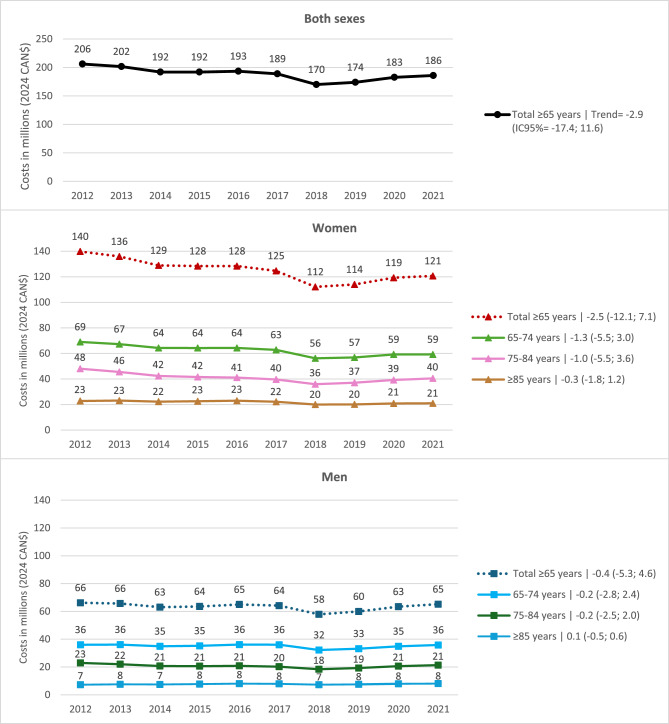
Overall actual claim costs of potentially inappropriate medications and by sex and age group among ≥65 years older adults in Quebec (2012-2021). Trend slopes (TS) and 95% confidence intervals (CI) are presented in the legend as follows: TS (95% CI). For example, for women in the 65-74 age group, -1.3 (-5.5; 3.0) indicates a TS of -1.3 with a 95% CI ranging from -5.5 to 3.0. The trend slope of -1.3 indicates that each year, the costs decreased by an average of $1.3 million

In 2021, the cost of medications and pharmaceutical services for ≥ 65 years represented $3,769,833,146. Deductibles totalled $328,684,766 (8.7%) and co-insurance $436,043,626 (11.6%), resulting in a direct cost to the RAMQ of $3,005,104,754 (79.7%) [25] (inflated to 2024: $3.44 billion). In 2021, the estimated total costs of PIM claims were $186 million, representing 5.4% of public expenditures in prescription medications in older adults. Compared to 2012, $186 million represents a 9.7% relative decrease from $206 million (Fig. 1). Although these costs revealed a downward trend, the decrease was not statistically significant, with a trend slope estimate (95% CI) of −2.9 (−17.4; 11.6). Specifically, costs went from $140 million to $121 million (−2.5 [−12.1; 7.1]) in women, and from $66 million to $65 million (−0.4 [−5.3; 4.6]) in men. Graphically, costs decreased early in the study period but increased after 2018, particularly for women. Visually, the decline in total costs seemed more noticeable for women than men across all age groups, although annual total costs consistently remained higher for women compared to men. For men, total costs remained stable across all age groups for the whole period. Within each sex, PIM claim costs were higher among younger enrollees.

The average per enrollee overall costs exhibited a more noticeable, although non-statistically significant, decreasing trend than the total costs (Table 2), with a decline from $179 to $119 (−33.5%). Among women, the average PIM costs per enrollee decreased from $216 in 2012 to $142 in 2021 (−34.3%), while for men, it decreased from $132 to $92 (−30.3%). Each age group followed a similar trend slope estimate, ranging from − 4 to −9.

**Table 2:** Average cost per enrollee and trend slopes of potentially inappropriate medication claims by sex and age group among ≥65 years older adults in Quebec (2012-2021)

	**Age group (years)**	**Average cost by year (2024 CAN $)**										**Trend slope (95% CI)**
		**2012**	**2013**	**2014**	**2015**	**2016**	**2017**	**2018**	**2019**	**2020**	**2021**	
**Women**	65-74	202	189	173	167	161	153	133	130	132	129	-8 (-22; 5)
	75-84	224	211	193	187	182	171	150	149	151	149	-9 (-24; 7)
	≥85	247	241	224	220	216	202	179	177	180	179	-9 (-24; 7)
	≥ 65	216	203	187	181	176	165	145	143	145	142	-9 (-23; 6)
**Men**	65-74	119	114	105	102	101	97	84	84	85	85	-4 (-12; 4)
	75-84	146	137	126	122	119	112	97	96	98	96	-6 (-16; 4)
	≥ 85	174	169	157	154	151	140	123	123	125	124	-6 (-17; 5)
	≥ 65	132	126	116	113	111	105	91	91	93	92	-5 (-13; 4)
**Overall**		179	169	156	151	147	139	121	119	121	119	-7 (-19; 5)

For each sex and age group, the average cost per enrollee was determined by dividing the total cost of PIM claims for the fiscal year by the number of full-time equivalent population for the calendar year. For example, the average cost of $202 per woman enrollee in the 65-74 age group in 2012 was calculated by dividing $69,019,334 (costs from April 1^st^, 2012, to March 31^st^, 2013) by 341,308 enrollees (full-time equivalent population from January 1^st^, 2012, to December 31^st,^ 2012)

The trend slope of -8 for women in the 65-74 group indicates that each year, the average cost per enrollee decreased by an average of $8

**Fig. 2:**
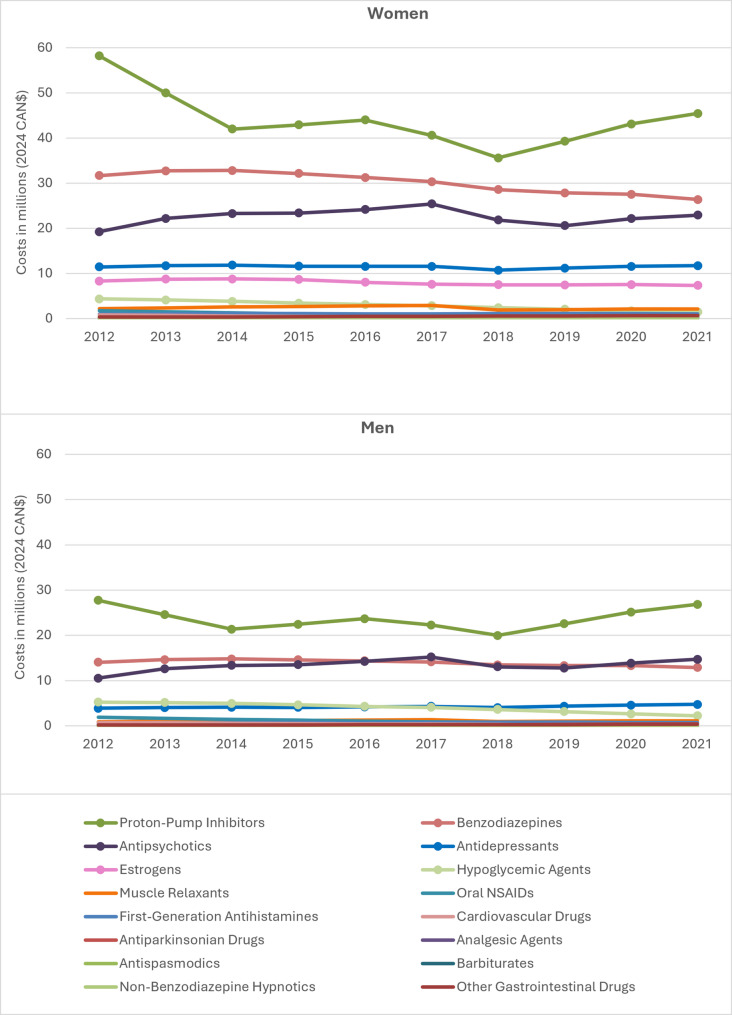
Total actual claim costs by potentially inappropriate medication class and sex among ≥65 years older adults in Quebec (2012-2021). NSAIDs, non-steroidal anti-inflammatory drugs. Note 1: This figure illustrates the costs of potentially inappropriate medication (PIM) classes among women and men over the study period. The most expensive classes are identified by marks on the series. Note 2: The graphs illustrate that the first four costliest PIM classes were the same for both sexes, but that the fifth most expensive class were estrogens for women and hypoglycemic agents for men

The four costliest PIM classes were PPIs, benzodiazepines, antipsychotics, and antidepressants (Fig. 2). The fifth costliest class was estrogens for women, and hypoglycemic agents for men. Most classes showed decreasing cost trends, though only the women’s slope for hypoglycemic agents was statistically significant (Table S3). Total costs for PPIs decreased from 2012 to 2021, with notable dips in 2014 and in 2018 for both sexes. Conversely, antipsychotics showed a slight, non-statistically significant increasing cost trend.

**Fig. 3:**
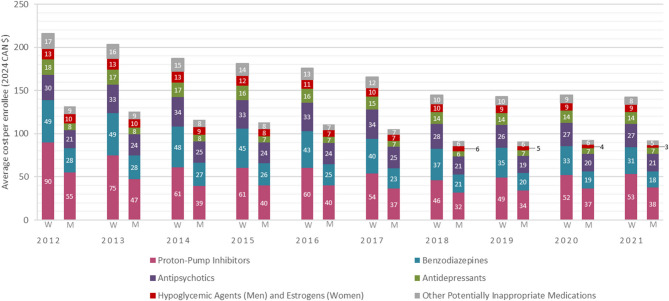
Average cost per enrollee by potentially inappropriate medication class and sex among ≥65 years older adults in Quebec (2012-2021). W: Women. M: Men. The five costliest potentially inappropriate medication (PIM) classes presented are proton-pump inhibitors, benzodiazepines, antipsychotics, antidepressants, hypoglycemic agents (for men) and estrogens (for women). Other PIMs include muscle relaxants, oral non-steroidal anti-inflammatory drugs, first-generation antihistamines, cardiovascular drugs, antiparkinsonian drugs, analgesic agents, antispasmodics, barbiturates, non-benzodiazepine hypnotics (Z-drugs) and other gastrointestinal drugs

Average costs per enrollee for the five costliest PIM classes remained consistently high and contributed to a mean of 93% of the total average cost per enrollee for all PIMs (Fig. 3). For women, the highest average costs per enrollee were for PPIs, followed by benzodiazepines, antipsychotics, antidepressants and estrogens. The order of these classes in terms of expenses remained consistent over the 10-year period. For men, the highest average costs per enrollee were also for PPIs, with benzodiazepines alternating with antipsychotics as the second costliest class from 2017. Hypoglycemic agents were the third costliest class until 2016, after which antidepressants became most costly per man enrollee. Despite the decrease (not statistically significant) of PPI costs among both sexes, prevalence of users increased. Average cost per enrollee of benzodiazepines statistically significantly decreased for both sexes (Table S5), reflecting the decreasing prevalence of users (Table 3).

**Table 3:** Number and proportion of enrolleesclaiming the costliest potentially inappropriate medication classes by sex and age group among ≥65 years older adults in Quebec (2012-2021)

**PIM class**	**Age (years)**	**Women n (%)**									
		**2012**	**2013**	**2014**	**2015**	**2016**	**2017**	**2018**	**2019**	**2020**	**2021**
Proton-Pump Inhibitors	65-74	55,933 (16.4)	63,090 (17.7)	69,254 (18.7)	75,483 (19.6)	81,101 (20.4)	83,955 (20.4)	81,961 (19.4)	89,051 (20.4)	94,529 (21.0)	98,675 (21.5)
	75-84	41,696 (19.4)	45,096 (20.9)	48,555 (22.2)	52,012 (23.4)	55,080 (24.3)	57,483 (24.8)	56,801 (23.7)	62,639 (25.1)	67,344 (25.9)	71,855 (26.4)
	≥85	20,195 (21.9)	22,955 (23.9)	25,271 (25.4)	27,516 (26.9)	29,895 (28.1)	31,528 (28.7)	30,517 (27.2)	32,998 (29.0)	34,530 (29.9)	36,052 (30.8)
	≥65	117,824 (18.2)	131,141 (19.6)	143,080 (20.8)	155,011 (21.9)	166,076 (22.7)	172,966 (23.0)	169,279 (21.8)	184,688 (23.1)	196,403 (23.8)	206,582 (24.3)
Benzodiazepines	65-74	106,871 (31.3)	108,147 (30.3)	108,036 (29.1)	107,373 (27.9)	106,376 (26.7)	103,233 (25.1)	99,196 (23.4)	95,963 (22.0)	91,362 (20.3)	88,083 (19.2)
	75-84	85,351 (39.8)	83,417 (38.6)	82,164 (37.5)	80,123 (36.1)	78,666 (34.7)	76,764 (33.1)	74,578 (31.1)	72,517 (29.1)	71,240 (27.4)	70,082 (25.8)
	≥85	38,522 (41.7)	39,480 (41.2)	40,098 (40.3)	39,516 (38.6)	40,029 (37.6)	39,414 (35.8)	38,125 (34.0)	36,513 (32.1)	35,516 (30.7)	33,974 (29.0)
	≥65	230,744 (35.6)	231,044 (34.6)	230,298 (33.4)	227,012 (32.0)	225,071 (30.8)	219,411 (29.1)	211,899 (27.3)	204,993 (25.7)	198,118 (24.0)	192,139 (22.6)
Antipsychotics	65-74	15,824 (4.6)	17,586 (4.9)	18,933 (5.1)	20,584 (5.4)	22,280 (5.6)	23,662 (5.8)	24,843 (5.9)	26,338 (6.0)	28,241 (6.3)	29,708 (6.5)
	75-84	14,949 (7.0)	15,664 (7.3)	16,139 (7.4)	16,465 (7.4)	17,047 (7.5)	17,568 (7.6)	17,921 (7.5)	18,562 (7.4)	20,240 (7.8)	21,299 (7.8)
	≥85	11,280 (12.2)	12,215 (12.7)	13,072 (13.1)	13,346 (13.0)	14,228 (13.4)	14,530 (13.2)	14,338 (12.8)	14,198 (12.5)	15,258 (13.2)	15,030 (12.8)
	≥65	42,053 (6.5)	45,465 (6.8)	48,144 (7.0)	50,395 (7.1)	53,555 (7.3)	55,760 (7.4)	57,102 (7.4)	59,098 (7.4)	63,739 (7.7)	66,037 (7.8)
Antidepressants	65-74	24,535 (7.2)	25,474 (7.1)	26,266 (7.1)	26,764 (7.0)	27,750 (7.0)	28,497 (6.9)	29,204 (6.9)	29,553 (6.8)	30,093 (6.7)	30,535 (6.6)
	75-84	13,780 (6.4)	13,763 (6.4)	13,918 (6.4)	14,140 (6.4)	14,455 (6.4)	14,853 (6.4)	15,202 (6.3)	15,809 (6.3)	16,394 (6.3)	17,081 (6.3)
	≥85	4,487 (4.9)	4,516 (4.7)	4,592 (4.6)	4,581 (4.5)	4,776 (4.5)	4,820 (4.4)	4,849 (4.3)	4,830 (4.2)	4,846 (4.2)	4,921 (4.2)
	≥65	42,802 (6.6)	43,753 (6.5)	44,776 (6.5)	45,485 (6.4)	46,981 (6.4)	48,170 (6.4)	49,255 (6.4)	50,192 (6.3)	51,333 (6.2)	52,537 (6.2)
Estrogens	65-74	29,719 (8.7)	30,563 (8.6)	30,722 (8.3)	30,489 (7.9)	29,941 (7.5)	27,804 (6.8)	27,063 (6.4)	26,328 (6.0)	25,670 (5.7)	25,201 (5.5)
	75-84	7,312 (3.4)	7,365 (3.4)	7,482 (3.4)	7,563 (3.4)	7,534 (3.3)	7,321 (3.2)	7,587 (3.2)	7,773 (3.1)	7,979 (3.1)	8,271 (3.0)
	≥85	976 (1.1)	1,012 (1.1)	1,062 (1.1)	1,076 (1.1)	1,140 (1.1)	1,111 (1.0)	1,159 (1.0)	1,185 (1.0)	1,192 (1.0)	1,281 (1.1)
	≥65	38,007 (5.9)	38,940 (5.8)	39,266 (5.7)	39,128 (5.5)	38,615 (5.3)	36,236 (4.8)	35,809 (4.6)	35,286 (4.4)	34,841 (4.2)	34,753 (4.1)
**PIM class**	**Age (years)**	**Men n (%)**									
		**2012**	**2013**	**2014**	**2015**	**2016**	**2017**	**2018**	**2019**	**2020**	**2021**
Proton-Pump Inhibitors	65-74	31,777 (10.5)	36,380 (11.4)	40,578 (12.2)	45,187 (13.1)	49,434 (13.8)	52,284 (14.1)	52,377 (13.6)	57,734 (14.5)	61,820 (15.1)	65,216 (15.4)
	75-84	22,825 (14.6)	25,354 (15.8)	27,739 (16.8)	30,365 (18.0)	32,440 (18.6)	34,632 (19.1)	34,815 (18.3)	39,298 (19.7)	42,598 (20.2)	45,975 (20.7)
	≥85	7,626 (18.3)	9,047 (20.3)	10,317 (21.8)	11,400 (22.8)	12,887 (24.2)	14,039 (24.9)	14,215 (24.1)	15,869 (25.9)	16,924 (26.7)	17,685 (27.2)
	≥65	62,228 (12.4)	70,781 (13.5)	78,634 (14.5)	86,952 (15.4)	94,761 (16.2)	100,955 (16.6)	101,407 (16.0)	112,901 (17.2)	121,342 (17.7)	128,876 (18.2)
Benzodiazepines	65-74	57,166 (18.8)	58,084 (18.3)	58,716 (17.7)	58,102 (16.9)	57,908 (16.2)	56,634 (15.3)	54,986 (14.3)	53,535 (13.5)	51,330 (12.5)	49,920 (11.8)
	75-84	40,098 (25.6)	39,559 (24.7)	39,334 (23.9)	38,797 (23.0)	38,380 (22.0)	37,653 (20.8)	36,649 (19.3)	35,936 (18.0)	35,577 (16.9)	35,026 (15.8)
	≥85	12,368 (29.6)	12,835 (28.8)	13,405 (28.3)	13,377 (26.8)	13,654 (25.6)	13,590 (24.1)	13,449 (22.8)	12,905 (21.1)	12,669 (20.0)	12,130 (18.6)
	≥65	109,632 (21.8)	110,478 (21.1)	111,455 (20.5)	110,276 (19.6)	109,942 (18.8)	107,877 (17.7)	105,084 (16.6)	102,376 (15.6)	99,576 (14.6)	97,076 (13.7)
Antipsychotics	65-74	10,987 (3.6)	12,286 (3.9)	13,604 (4.1)	14,568 (4.2)	16,078 (4.5)	17,187 (4.6)	18,232 (4.8)	19,512 (4.9)	20,939 (5.1)	22,350 (5.3)
	75-84	8,807 (5.6)	9,331 (5.8)	9,845 (6.0)	10,146 (6.0)	10,592 (6.1)	11,156 (6.2)	11,204 (5.9)	11,721 (5.9)	13,108 (6.2)	13,771 (6.2)
	≥85	4,103 (9.8)	4,566 (10.2)	4,928 (10.4)	5,152 (10.3)	5,565 (10.4)	5,676 (10.1)	5,854 (9.9)	5,980 (9.8)	6,410 (10.1)	6,453 (9.9)
	≥65	23,897 (4.8)	26,183 (5.0)	28,377 (5.2)	29,866 (5.3)	32,235 (5.5)	34,019 (5.6)	35,290 (5.6)	37,213 (5.7)	40,457 (5.9)	42,574 (6.0)
Antidepressants	65-74	9,487 (3.1)	9,902 (3.1)	10,258 (3.1)	10,616 (3.1)	11,357 (3.2)	11,845 (3.2)	12,304 (3.2)	12,952 (3.3)	13,378 (3.3)	13,923 (3.3)
	75-84	4,823 (3.1)	4,892 (3.1)	4,984 (3.0)	5,081 (3.0)	5,266 (3.0)	5,477 (3.0)	5,825 (3.1)	6,086 (3.0)	6,349 (3.0)	6,697 (3.0)
	≥85	1,064 (2.5)	1,126 (2.5)	1,234 (2.6)	1,230 (2.5)	1,259 (2.4)	1,335 (2.4)	1,354 (2.3)	1,325 (2.2)	1,416 (2.2)	1,411 (2.2)
	≥65	15,374 (3.1)	15,920 (3.0)	16,476 (3.0)	16,927 (3.0)	17,882 (3.1)	18,657 (3.1)	19,483 (3.1)	20,363 (3.1)	21,143 (3.1)	22,031 (3.1)
Hypoglycemic Agents	65-74	18,030 (5.9)	17,637 (5.5)	17,021 (5.1)	16,352 (4.7)	15,534 (4.3)	14,356 (3.9)	12,318 (3.2)	10,349 (2.6)	8,476(2.1)	7,080 (1.7)
	75-84	9,303 (5.9)	8,715 (5.4)	8,210 (5.0)	7,634 (4.5)	7,218 (4.1)	6,722 (3.7)	6,016 (3.2)	5,266 (2.6)	4,456 (2.1)	3,909 (1.8)
	≥85	1,574 (0.7)	1,552 (0.6)	1,495 (0.6)	1,430 (0.6)	1,310 (0.5)	1,227 (0.7)	1,082 (0.7)	1,014 (0.7)	879 (0.6)	735 (0.5)
	≥65	28,907 (5.8)	27,904 (5.3)	26,726 (4.9)	25,416 (4.5)	24,062 (4.1)	22,305 (3.7)	19,416 (3.1)	16,629 (2.5)	13,811 (2.0)	11,724 (1.7)

In the sensitivity analysis (2012 to 2018), all slope estimates for costs were not statistically significant (Table S2). Similarly, the analysis considering nominal PIM costs yielded results comparable to those for actual costs (Supplementary files).

## Discussion

Our findings demonstrate that PIMs continue to impose a substantial cost on public healthcare, with expenses remaining relatively stable over the past decade. Average per enrollee costs illustrate the evolution of PIM expenditures while also acknowledging for the increasing number of older adults due to aging population. Women, who outnumbered men, incurred higher total and per enrollee costs yet exhibited a more pronounced decreasing trend than men (in absolute and relative values). PPIs and benzodiazepines were the PIM classes that most significantly impacted the economic burden.

Cost analyses to evaluate financial burden of specific medications in Canada are scarce, particularly regarding recent estimates attributable to PIMs. PIM-related costs across Canada were estimated at $419 million in 2013; $75/enrollee [12] (inflated to 2024: $548 million; $98/enrollee), and at $1 billion in 2021; $140/enrollee [16] (inflated to 2024: $1.1 billion; $150/enrollee). In comparison, our data for Quebec shows $202 million; $169/enrollee, and $186 million; $119/enrollee for the same years, respectively. Our analysis included different PIM classes than those in these studies. The first study relied on the 2012 version of the Beers criteria, which did not include PPIs [26], a major contributor to PIM costs. The most recent study defined PIMs by combining hand-picked PIMs from various lists and included opioids, gabapentinoids, and cholinesterase inhibitors not included in the Beers criteria [16]. By removing these three medications from their study, Canadian PIM costs fall to $747 million; $104/enrollee [16] (inflated to 2024: $801 million; $112/enrollee). Moreover, there was no mention of exclusion of parenteral forms or consideration of concomitances (e.g. PPIs-NSAIDs), and both deductible and co-insurance costs paid by enrollees were included, likely increasing cost estimates compared to ours. Nonetheless, this could suggest that Quebec’s PIM expenditures were considerably higher than in the rest of Canada. Compared to national projections indicating a 33% decline in inflation-adjusted PIM costs between 2013 and 2021 [16], our data also point to a decreasing trend (−9.7%), though the absolute costs and slope differ due to the factors outlined above.

Other studies conducted in various countries have analyzed PIM reimbursement data among adults ≥ 65 years [27, 28]. Similar to our findings, prevalences were mainly attributed to PPIs and benzodiazepines in Lithuania (2015) [29], France (2017) [28], and United States (Medicare Part D) (2014–2018) [30]. A decrease in benzodiazepine use was also found in France when compared to anterior studies [28]. However, while our results showed higher PIM costs and prevalent use among women, these findings where not observed for prevalence in Lithuania [29] or for costs in France [28]. Reimbursement policies vary greatly across countries, including differences in exemptions or cost-sharing mechanism [31]. Differences in healthcare systems, disease epidemiology, prescribing habits and PIM identification tools (e.g. 2003, 2015, and 2019 Beers criteria, EU(7)-PIM list, PRISCUS list, REMEDI[e]S, and STOPP criteria) may contribute to these disparities. For instance, in the US, benzodiazepines were initially excluded from Medicare Part D coverage, but were added in 2013 [32]. Prescriptions are now valid for six months and can be refilled up to five times within that period. After six months, a new prescription is required, and this cycle can be repeated. Following this change, rates of benzodiazepines-related overdoses and fall-related injuries increased in older adults [33]. In France, benzodiazepine prescriptions are subject to strict time limits and regulatory conditions, including a maximum duration of 12 weeks for anxiolytics and four weeks for hypnotics [34]. In Lithuania, regulatory changes introduced in 2021 limited benzodiazepine prescriptions to a maximum of 30 days with a maximum of a 10-day period for dispensing, and a psychotropic medication prescription record [35]. These regulations aimed to limit long-term use and reduce the possibility of prescription accumulation across multiple prescribers. These systemic differences likely contribute to variations in PIM use and associated costs and may inform the development of more effective policy levers.

For both sexes, the most expensive PIM classes were PPIs and benzodiazepines during the whole study period. Several regulatory measures likely contributed to the decrease in PPI costs, despite an increase in the proportions of users. Over the decade, no new generic PPI was reimbursed by the public drug plan [36, 37]. However, a maximum payable price (maximal amount per pill that the public drug insurance agrees to reimburse) was introduced in 2013 [38], and was reduced in 2015 [39]. It is also possible that shorter treatment durations and fewer claims per individual by year contributed to the increase in PPI use prevalence, despite the decline in annual costs, suggesting potential changes in patterns of use. On the other side, the implementation of exception codes for PPIs in 2017, restricting their reimbursement to cases where the prescriber provided a valid indication associated with a specific code to limit their long-term use [14], likely led to an initial decrease in both costs and prevalence. However, this effect did not seem to persist, consistent with a recent Quebec study that showed exception codes were ineffective to prevent inappropriate PPI prescribing [40]. Since 2020, Quebec pharmacists can initiate PPIs and renew exception codes, which could also have contributed to their initiation and maintenance of these treatments [41, 42]. This pattern suggests that these regulatory measures may have had only a temporary impact and could potentially be circumvented by prescribers. One possible explanation is that PPI use has become normalized in clinical practice, and prescribers may feel ill-equipped or lack confidence in managing patients with alternative therapeutic or non-pharmacological options, particularly in complex older adults.

For benzodiazepines, both costs per enrollee and prevalence of use have decreased considerably, consistent with the trend observed in the last years [43, 44]. This decline mirrors awareness efforts by various organizations, including the Canadian Medication Appropriateness and Deprescribing Network and Beers criteria updates [9, 10, 13]. However, part of this decrease could also result from a shift towards other PIMs, such as antipsychotics and antidepressants. Interestingly, a shift towards Z-drugs was not observed in a study conducted across all age groups in Canada from 2016 to 2022 [45]. Despite these efforts, benzodiazepines and PPIs remain major contributors to the overall burden of PIMs in the older population. These findings underscore the need for sustained and multifaceted interventions, including patient and prescriber education, better enforcement of reimbursement restrictions, and regular medication reviews, to optimize medication use and reduce inappropriate prescribing in older adults.

From the public payor perspective, the economic burden of PIMs is likely much higher, as indirect costs such as hospitalizations or outpatient visits for adverse effects substantially add to medication-related expenses [46, 47]. Based on the RAMQ medication costs for adults aged ≥ 65 in 2021 [25] (inflated to 2024: $3.44 billion), the weight of PIM direct costs represented around 5.4%. This constitutes a significant savings opportunity, considering both direct and indirect costs. While some studies have attempted to quantify indirect costs, such as those associated with increased healthcare utilization, a comprehensive assessment of the full societal impact remains limited. In Canada, the estimated incremental health care utilization cost associated with PIMs was $1.4 billion in 2013 ($1.8 billion inflated to 2024). A retrospective study in Ontario using the STOPP/START criteria estimated a total cost of $1.5 billion (inflated to 2024) for newly prescribed medications (not necessarily PIMs), including hospitalizations and emergency department visits attributable to PIMs [48].

### Strengths and limitations

To our knowledge, this is the first cost analysis conducted over a decade and encompassing nearly the entire Quebec population aged ≥ 65 with highly reliable data. Nonetheless, our study has limitations. The lack of clinical indications and diagnoses in administrative data prevented confirmation of the inappropriateness of the included medication, as clinical contexts could justify the use of some listed PIMs. However, we implemented several conditions to limit possible overestimates of PIM claims and costs. Besides, due to the absence of individual data notably about socioeconomic status and comorbidities, multivariate analysis could not be performed. However, we conducted stratified analysis for key variables such as sex and age. We also did not test for the assumption of independent observations, which could have led us to false statistically significant results and unreliable estimations. Additionally, our study focused exclusively on direct PIM costs and did not assess the additional indirect costs associated with PIM use in older adults, underestimating costs borne by public funds. These indirect costs were beyond the scope of the study, which relied on administrative medication claims data without linked clinical outcomes. Future studies incorporating comprehensive hospitalizations, adverse drug reactions, increased use of healthcare services, and productivity losses are needed to better capture the overall societal impact. A more complete understanding of both direct and indirect costs will help policymakers and clinicians appreciate the true magnitude of PIM-related economic burden and guide the design of targeted interventions to optimize medication use in older adults.

## Conclusion

This study highlights the stable 10-year trend in PIM costs among older women and men in Quebec. Optimising prescribing practices and actively implementing effective deprescribing strategies are essential not only for reducing PIM costs but also for improving individuals’ health and reducing indirect PIM costs. Further research should focus on estimating the societal impact associated with PIM use to support the development of sustainable strategies promoting optimal medication use in this population. These findings emphasize the need of ongoing monitoring and evaluation of PIM costs to better assess the effectiveness of deprescribing initiatives and other regulatory measures.

## Supplementary Information

Supplementary Material 1.

## Data Availability

The medication data that support the findings of this study are available on request ($) from the Régie de l’assurance maladie du Québec. Restrictions apply to the availability of these data, which were used under license for the present study, and we cannot share them. We also used publicly available data, accessible at St@tRAMQ: https://www4.prod.ramq.gouv.qc.ca/Ist/CD/CDF_DifsnInfoStats/CDF1_CnsulInfoStatsCNC_iut/DifsnInfoStats.aspx? LANGUE=en-CA.
