# Supplementary material for: Cost trends of potentially inappropriate medications among older adults between 2012 and 2021 in Quebec, Canada: a population-based repeated cross-sectional study
Source: BMC Geriatr. 2025 Aug 2;25:583. doi: 10.1186/s12877-025-06235-7 (PMC12318379; doi:10.1186/s12877-025-06235-7)
Supplement: Supplementary file 1 — Supplementary Material 1. [file 12877_2025_6235_MOESM1_ESM.docx]

**SUPPLEMENTARY FILES**

**Table S1: Included potentially inappropriate medication on Beers criteria list (2015 and 2019 versions)**

| **Class** | **Medication** | **Common Denomination Drug Code** |
| --- | --- | --- |
| Analgesic Agents | meperidine  pentazocine | 05603-46412  44528 |
| Antidepressants | amitriptyline  amoxapine  clomipramine  desipramine  doxepine^1^  imipramine  nortriptyline  paroxetine  protriptyline  trimipramine | 00429-00442-46011  43696  14781  02522  03198  04784  06578  47061  08294  09906 |
| Antiparkinsonian Drugs | benztropine (oral only)  trihexyphenidyl | 34323  09828 |
| Antipsychotics | droperidol  fluphenazine  flupentixol  haloperidol  loxapine  pimozide  pipothiazine  thiopropazate  thioproperazine  thiothixene  thioridazine  zuclopenthixiol  pericyazine  aripiprazole  asenapine  clozapine  olanzapine  paliperidone  quetiapine  risperidone  ziprasidone  lurasidone  brexpiprazole | 03250-33855  04056-04069-34284  41863-43202  04394-43540-43826-46292  34219-37612-40745  33465  41707  09555  09568  09620-10400  09594  47136-47137-47138  07150  47801  47921  45580  46318-47197  47708-47861  47267-46413  46156-47052-47278  47717  47939  48153 |
| Antispasmodics (only topical patch and oral) | atropine  belladone  clidinium  dicyclomine  hyoscyamine  propantheline  scopolamine  hyoscine | 00689-10881-18829-45466-46721-39627-11245-46038  ~~4~~6307  46166  02756-46685  46205  08203  08814-08827-34687-46109-46827  46074 |
| Barbiturates | amobarbital  butabarbital  butobarbital  butalbital  mephobarbital  pentobarbital  pentabarbital  phenobarbitalsecobarbital | 00468-00481  01157-01170  34349  46012-46013  05642  07124  46072  07345-17906-19128-46172-46338-46558-46118  08853 |
| Benzodiazepines (oral only) | alprazolam  bromazepam  lorazepam  oxazepam  temazepam  triazolam  clobazam  clorazepate  chlordiazepoxide  clonazepam  diazepam  flurazepam  nitrazepam | 43501  43488  37950-46440  06786  41590  39029  45591  14768  01807  37872  02717-46161  04095-46818  42045 |
| Cardiovascular Drugs | dipyridamole (oral short acting, excluding the extended-release combination with aspirin)  ticlopidine  guanfacine  methyldopa  disopyramide  dronedarone  digoxine (>6 mg/day)  nifedipine | 03094-46077-47365  45617  47979  06136  37911-38080  47804  02847  42708-46388-46469-47751 |
| First-Generation Antihistamines (oral only) | brompheniramine  chlorpheniramine  clemastine  cyproheptadine  dexbrompheniramine  dexchlorpheniramine  dimenhydrinate  diphenhydramine  doxylamine  hydroxyzine  ketotifen  meclizine  phenyltoloxamine  promethazine  pyrilamine (2019 version only)  trimeprazine  triprolidine | 01131-46058  01885-45575-46107-46364-46730  39068  02379  46257  02639  02938-46212  03107-46381-46148-46073  46045-46140-47131-48207  04706  45555-46752  05512-46079  46255  08177-34336-46439  46323  09841  09945 |
| Hypoglycemic Agents^2^ | chlorpropramide  glyburide  glimepiride (2019 version only)  human insulin  insulin lispro  insulin aspart  insulin glulisine | 01937  04264  46799-47652-47427  44489  47206  47424  47749 |
| Muscle Relaxants | carisoprodol  chlorzoxazone  cyclobenzaprine  methocarbamol  orphenadrine | 46167  46143-46421  38873-46516  06019-46098-46183-46871-47570  06734-06747-46094-46254 |
| Non-Benzodiazepine Hypnotics | zolpidem  zaleplon  zopiclone  eszopiclone | 47912  46668  46047  00028 |
| Estrogens (only in women and only those with systemic effect) | estradiol  estrogens (esterified)  biological conjugated estrogens  synthetic conjugated estrogens  ethinylestradiol  estradiol-17B  diethylstilbesterol  estradiol 0,1 %  estradiol hemihydrate  estriol  conjugated estrogens  estropipate  megestrol  mestranol  oestrone | 47759-06643-45022-47752-10842-46121-10868-45360  43072  45582-47395  45583  03627-46460-47053-03640-46850-03666-47557-47869-33491-03705-47041-47054-47485-03679-46414-46697-45477  46242-34232-45488-46417-46766-47111-46489-47748-47374  02795-02808-46696  47947  46840  46557  48165-43059  47031  38483  05759-05785-05798  33556 |
| Oral NSAIDs^3^ | indomethacin^4^  ketorolac^4^  aspirin (>325 mg/day)  diclofenac  diflunisal  etodolac  fenoprofen calcium  ibuprofen  ketoprofen  mefenamic  meloxicam  nabumetone  naproxen  oxaprozine  piroxicam  sulindac  flurbiprofen  tiaprofenic | 04810  46006-47066  00143-38184-46232-46353  41694-47078  43150  46256-47122  33803  04745-46654-47506  38691  44359  47385  47084  46152-46335-46626-19752  46347  42019-46638  40381  44749  45407 |
| Other Central Nervous System Drugs (excluded) | ergoloids  isoxsuprine | 46180  46025 |
| Other Gastrointestinal Drugs (oral only) | mineral oil  metoclopramide | 04498-42539-43657-46017  18595-46700 |
| Proton-Pump Inhibitors^5^ (oral only) | dexlansoprazole  esomeprazole  lansoprazole  omeprazole  pantoprazole  rabeprazole | 47850  46761-47418  47140  45519-46713-47146-47622  46365-47234-47616  47432 |
| Thyroid Drugs (excluded) | thyroid  thyroid extract | 09659  46590 |

NSAIDs, non-steroidal anti-inflammatory drugs

^1^Doxepin was considered inappropriate only for dosage strengths of 10 mg, 25 mg, 50 mg, 75 mg, 100 mg and 150 mg per tablet.

^2^Rapid-acting insulins were considered inappropriate in the absence of concomitant use with intermediate- or long-acting insulins (44164, 45531, 47426, 47536, 47586, 47615, 48163, 48174)

^3^Except for indomethacin and ketorolac, oral NSAIDs were considered inappropriate if the treatment period exceeded 30 days in the absence of concomitant use with a proton-pump inhibitor or misoprostol

^4^Parental (excepted ophthalmic) and suppository forms were included as PIMs.

^5^Proton-pump inhibitors were considered inappropriate if the treatment period exceeded 8 weeks in the absence of concomitant use with oral NSAIDs or corticosteroids.

**Table S2: Trend slopes and confidence intervals for claim costs of potentially inappropriate medication by sex and age group among ≥65 years older adults in Quebec (2012-2018)**

|  |  | Trend slopes (95% CI) | |
| --- | --- | --- | --- |
| Sex | **Age group (year)** | **Actual costs* (2024 CAN $)** | **Nominal costs* (not adjusted for inflation)** |
| Women | 65-74 | -1.69 (-5.44; 2.06) | -0.61 (-3.44; 2.21) |
|  | 75-84 | -1.76 (-3.82; 0.30) | -0.91 (-2.50; 0.68) |
|  | ≥85 | -0.34 (-1.98; 1.31) | -0.02 (-1.22; 1.19) |
|  | ≥65 | -3.79 (-10.80; 3.22) | -1.54 (-6.81; 3.73) |
| Men | 65-74 | -0.37 (-2.88; 2.14) | 0.11 (-1.77; 1.99) |
|  | 75-84 | -0.61 (-1.78; 0.56) | -0.24 (-1.14; 0.65) |
|  | ≥85 | 0.05 (-0.54; 0.64) | 0.12 (-0.30; 0.55) |
|  | ≥65 | -0.93 (-5.04; 3.18) | -0.01 (-3.09; 3.06) |

*In million

Note 1: Trend slopes were calculated for 2012 to 2018, period corresponding to the application of the American Geriatrics Society’s 2015 version of the Beers criteria (9) before the application of the 2019 version (10).

Note 2: The trend slope of -1.69 for women in the 65-74 group indicates that each year, the costs decreased by an average of $1.69 million for the 2012 to 2018 period.

**Table S3: Trend slope estimates and confidence intervals for claim costs* of all potentially inappropriate medication classes by sex among ≥65 years older adults in Quebec (2012-2021)**

|  | | Trend slope (95% CI) | |
| --- | --- | --- | --- |
| Serie | | **2024 $ value** | **Nominal value** |
| Women | Proton-Pump Inhibitors | -1.22 (-11.64; 9.19) | -0.40 (-8.80; 8.00) |
|  | Benzodiazepines | -0.73 (-2.44; 0.98) | -0.22 (-1.43; 0.98) |
|  | Antipsychotics | 0.10 (-3.50; 3.69) | 0.35 (-2.37; 3.07) |
|  | Antidepressants | -0.03 (-0.67; 0.62) | 0.12 (-0.40; 0.64) |
|  | Estrogens | -0.17 (-0.74; 0.41) | -0.04 (-0.46; 0.39) |
|  | Hypoglycemic Agents | -0.33 (-0.42; -0.24) | -0.23 (-0.33; -0.13) |
|  | Muscle Relaxants | -0.05 (-0.71; 0.62) | -0.01 (-0.53; 0.51) |
|  | Oral NSAIDs | -0.12 (-0.35; 0.11) | -0.08 (-0.25; 0.09) |
|  | First-Generation Antihistamines | 0.01 (-0.08; 0.10) | 0.02 (-0.05; 0.09) |
|  | Cardiovascular Drugs | -0.07 (-0.15; 0.00) | -0.05 (-0.11; 0.01) |
|  | Antiparkinsonian Drugs | 0.04 (-0.06; 0.13) | 0.03 (-0.05; 0.11) |
|  | Analgesic Agents | -0.01 (-0.04; 0.03) | -0.01 (-0.03; 0.02) |
|  | Antispasmodics | 0.02 (-0.07; 0.10) | 0.02 (-0.06; 0.09) |
|  | Barbiturates | 0.01 (-0.03; 0.04) | 0.01 (-0.02; 0.03) |
|  | Non-Benzodiazepines Hypnotics | 0.00 (0.00; 0.01) | 0.00 (0.00; 0.01) |
|  | Other Gastrointestinal Drugs | 0.04 (-0.01; 0.08) | 0.04 (0.00; 0.08) |
| Men | Proton-Pump Inhibitors | -0.04 (-5.11; 5.03) | 0.28 (-3.92; 4.49) |
|  | Benzodiazepines | -0.19 (-0.91; 0.54) | 0.02 (-0.47; 0.51) |
|  | Antipsychotics | 0.26 (-1.90; 2.42) | 0.37 (-1.26; 2.00) |
|  | Antidepressants | 0.08 (-0.19; 0.35) | 0.12 (-0.12; 0.35) |
|  | Hypoglycemic Agents | -0.35 (-0.72; 0.03) | -0.23 (-0.58; 0.11) |
|  | Muscle Relaxants | 0.01 (-0.31; 0.32) | 0.02 (-0.22; 0.26) |
|  | Oral NSAIDs | -0.14 (-0.38; 0.10) | -0.09 (-0.27; 0.08) |
|  | First-Generation Antihistamines | 0.01 (-0.06; 0.09) | 0.02 (-0.04; 0.08) |
|  | Cardiovascular Drugs | -0.08 (-0.17; 0.01) | -0.06 (-0.14; 0.02) |
|  | Antiparkinsonian Drugs | 0.04 (-0.06; 0.13) | 0.03 (-0.05; 0.11) |
|  | Analgesic Agents | -0.00 (-0.01; 0.01) | 0.00 (-0.01; 0.01) |
|  | Antispasmodics | 0.01 (-0.04; 0.07) | 0.01 (-0.04; 0.06) |
|  | Barbiturates | 0.01 (-0.01; 0.02) | 0.01 (0.00; 0.02) |
|  | Non-Benzodiazepines Hypnotics | 0.00 (-0.00; 0.01) | 0.00 (0.00; 0.01) |
|  | Other Gastrointestinal Drugs | 0.02 (-0.00; 0.04) | 0.02 (0.00; 0.03) |

*In million

NSAIDs, non-steroidal anti-inflammatory drugs

The trend slope of -1.22 for proton-pump inhibitors among women indicates that each year, the costs decreased by an average of $1.22 million.

**Figure S1: Overall claim costs in nominal value of potentially inappropriate medications and by sex and age group among ≥65 years older adults in Quebec (2012-2021)**


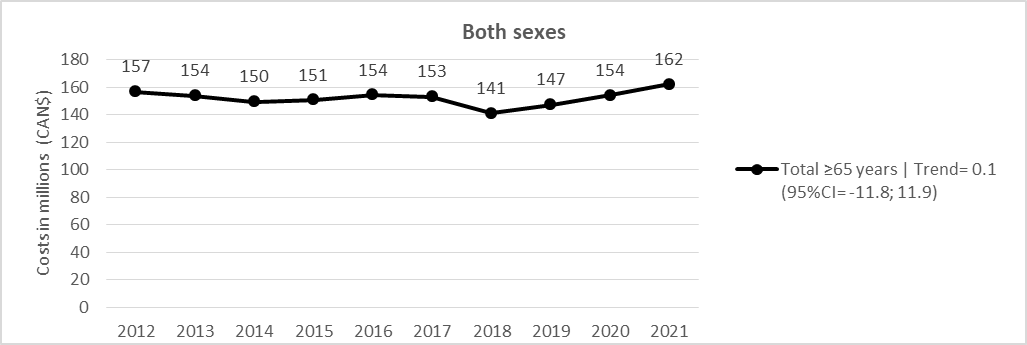

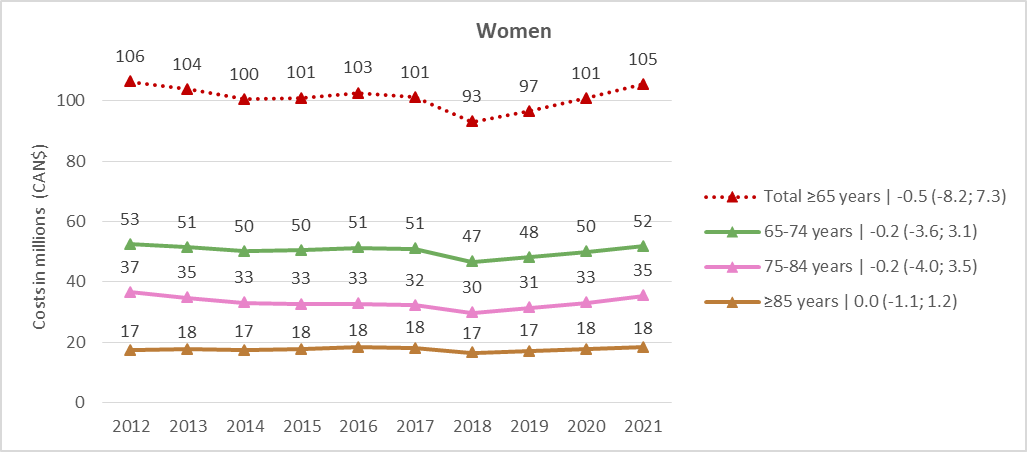

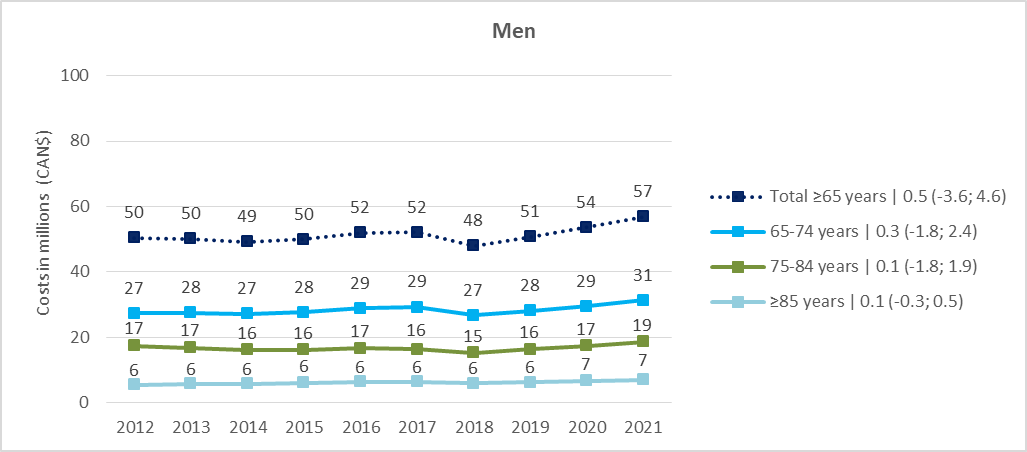


Trend slopes (TS) and 95% confidence intervals (CI) are presented in the legend as follows: TS (95% CI). For example, for women in the 65-74 age group, -0.2 (-3.6; 3.1) indicates a TS of -0.2 with a 95% CI ranging from -3.6 to 3.1. The trend slope of -0.2 indicates that each year, the costs decreased by an average of $0.2 million.

**Table S4: Average cost per enrollee in nominal value and trend slope estimates of potentially inappropriate medication claims by sex and age group (2012-2021)**

|  | Age group (years) | Average cost by year ($) | | | | | | | | | | Trend slope (95% CI) |
| --- | --- | --- | --- | --- | --- | --- | --- | --- | --- | --- | --- | --- |
|  |  | **2012** | **2013** | **2014** | **2015** | **2016** | **2017** | **2018** | **2019** | **2020** | **2021** |  |
| Women | 65-74 | 154 | 144 | 135 | 131 | 129 | 124 | 110 | 110 | 111 | 113 | -5 (-15; 5) |
|  | 75-84 | 170 | 161 | 151 | 147 | 145 | 139 | 124 | 126 | 128 | 130 | -5 (-17; 7) |
|  | ≥85 | 188 | 184 | 175 | 173 | 172 | 164 | 148 | 149 | 152 | 156 | -4 (-16; 8) |
|  | ≥ 65 | 164 | 155 | 146 | 142 | 140 | 134 | 120 | 121 | 122 | 124 | -5 (-16; 6) |
| Men | 65-74 | 90 | 87 | 82 | 80 | 81 | 79 | 70 | 71 | 72 | 74 | -2 (-8; 4) |
|  | 75-84 | 111 | 105 | 98 | 96 | 95 | 91 | 80 | 82 | 83 | 84 | -3 (-11; 5) |
|  | ≥ 85 | 132 | 129 | 122 | 121 | 120 | 113 | 102 | 104 | 106 | 108 | -3 (-12; 5) |
|  | ≥ 65 | 100 | 96 | 90 | 89 | 89 | 86 | 76 | 77 | 78 | 80 | -2 (-9; 4) |
| Overall |  | 136 | 129 | 121 | 119 | 117 | 113 | 100 | 101 | 102 | 104 | -4 (-13; 5) |

Note 1: For each sex and age group, the average cost per enrollee was determined by dividing the total cost of PIM claims for the fiscal year by the number of full-time equivalent population for the calendar year. For example, the average cost of $154 per woman enrollee in the 65-74 age group in 2012 was calculated by dividing $52,516,579 (costs from April 1^st^, 2012, to March 31^st^, 2013) by 341,308 enrollees (full-time equivalent population from January 1^st^, 2012, to December 31^st^, 2012).

Note 2: The trend slope of -5 for women in the 65-74 age group indicates that each year, the average cost per enrollee decreased by an average of $5.

Costs in millions (CAN$)

**Figure S2: Total claim costs in nominal value by potentially inappropriate medication class and sex among ≥65 years older adults in Quebec (2012-2021)**

NSAIDs, non-steroidal anti-inflammatory drugs

This figure illustrates the costs of the most expensive PIM classes among women and men over the study period. The most expensive classes are identified by marks on the series.

**
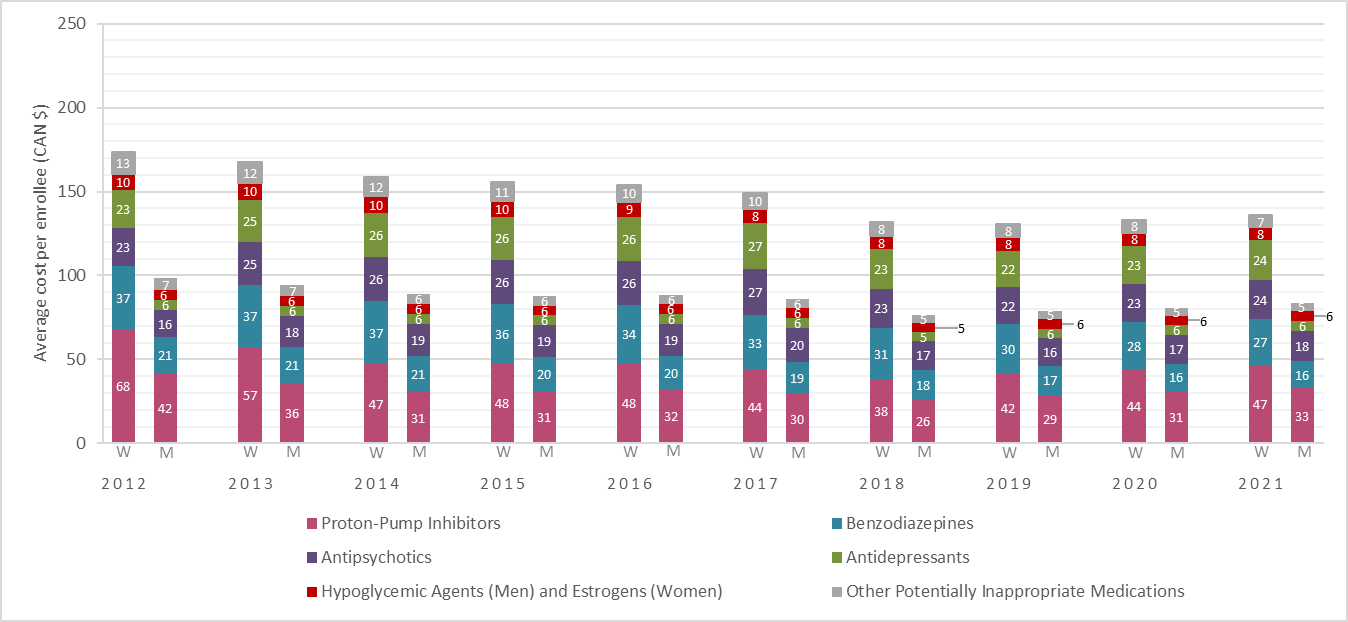
**

**Figure S3: Average cost per enrollee in nominal value by potentially inappropriate medication class and sex among ≥65 years older adults in Quebec (2012-2021)**

W: Women

M: Men

The five costliest potentially inappropriate medication (PIM) classes presented are proton-pump inhibitors, benzodiazepines, antipsychotics, antidepressants, hypoglycemic agents (for men) and estrogens (for women). Other PIMs include muscle relaxants, oral non-steroidal anti-inflammatory drugs, first-generation antihistamines, cardiovascular drugs, antiparkinsonian drugs, analgesic agents, antispasmodics, barbiturates, non-benzodiazepine hypnotics (Z-drugs) and other gastrointestinal drugs.

**Table S5: Trend slope estimates and confidence intervals for average costs per enrollee by potentially inappropriate medication classes and sex among ≥65 years older adults in Quebec (2012-2021)**

|  | | Trend slope (95% CI) | |
| --- | --- | --- | --- |
| Serie | | **2024 $ value** | **Nominal value** |
| Women | Proton-Pump Inhibitors | -3.6 (-19.0; 11.8) | -2.1 (-14.2; 10.0) |
|  | Benzodiazepines | -2.2 (-4.1; -0.2) | -1.3 (-2.7; 0.2) |
|  | Antipsychotics | -0.7 (-5.6; 4.1) | -0.2 (-4.0; 3.5) |
|  | Antidepressants | -0.5 (-1.4; 0.3) | -0.2 (-0.9; 0.4) |
|  | Estrogens | -0.5 (-1.4; 0.3) | -0.3 (-0.9; 0.3) |
|  | Other Potentially Inappropriate Medications | -1.0 (-1.6; -0.4) | -0.7 (-1.1; -0.2) |
| Men | Proton-Pump Inhibitors | -1.7 (-10.8; 7.4) | -0.9 (-8.1; 6.4) |
|  | Benzodiazepines | -1.2 (-2.2; -0.2) | -0.7 (-1.4; 0.1) |
|  | Antipsychotics | -0.4 (-4.1; 3.4) | 0.0 (-2.9; 2.8) |
|  | Antidepressants | -0.1 (-0.6; 0.3) | 0.0 (-0.4; 0.3) |
|  | Hypoglycemic Agents | -0.8 (-1.1; -0.6) | -0.6 (-0.9; -0.3) |
|  | Other Potentially Inappropriate Medications | -0.5 (-0.9; 0.0) | -0.3 (-0.6; 0.0) |

The trend slope of -3.6 for proton-pump inhibitors among women indicates that each year, the average cost per enrollee decreased by an average of $3.6.
